# The give and take of Arctic greening: differential responses of the carbon sink-to-source threshold to light and temperature in tussock tundra may be influenced by vegetation cover

**DOI:** 10.1038/s42003-024-06600-z

**Published:** 2024-08-06

**Authors:** Elizabeth Min, Natalie T. Boelman, Laura Gough, Jennie R. McLaren, Edward B. Rastetter, Rebecca J. Rowe, Adrian Rocha, Matthew H. Turnbull, Kevin L. Griffin

**Affiliations:** 1 Department of Earth and Environmental Science, Columbia University, New York City, NY USA; 2 Lamont-Doherty Earth Observatory, Columbia University, Palisades, NY USA; 3 Department of Biological Sciences, Towson University, Towson, MD USA; 4 Department of Biological Sciences, The University of Texas at El Paso, El Paso, TX USA; 5 The Ecosystems Center, Marine Biological Laboratory, Woods Hole, MA USA; 6 Department of Natural Resources and the Environment, University of New Hampshire, Durham, NH USA; 7 Department of Biological Sciences, University of Notre Dame, Notre Dame, IN USA; 8 School of Biological Sciences, University of Canterbury, Christchurch, New Zealand; 9 Department of Ecology, Evolution and Environmental Biology, Columbia University, New York City, NY USA

**Keywords:** Plant physiology, Climate-change ecology

## Abstract

A significant warming effect on arctic tundra is greening. Although this increase in predominantly woody vegetation has been linked to increases in gross primary productivity, increasing temperatures also stimulate ecosystem respiration. We present a novel analysis from small-scale plot measurements showing that the shape of the temperature- and light-dependent sink-to-source threshold (where net ecosystem exchange (NEE) equals zero) differs between two tussock tundra ecosystems differing in leaf area index (LAI). At the higher LAI site, the threshold is exceeded (i.e the ecosystem becomes a source) at relatively higher temperatures under low light but at lower temperatures under high light. At the lower LAI site, the threshold is exceeded at relatively lower temperatures under low light but at higher temperatures under high light. We confirmed this response at a single site where LAI was experimentally increased. This suggests the carbon balance of the tundra may be sensitive to small increases in temperature under low light, but that this effect may be significantly offset by increases in LAI. Importantly, we found that this LAI effect is reversed under high light, and so in a warming tundra, greater vegetation cover could have a progressively negative effect on net carbon uptake.

## Abstract (web summary)

The tundra carbon balance is sensitive to small temperature increases at low light, but the effect is offset by increases in leaf area index, or reversed at high light. With warming, greater vegetation cover could negatively affect net carbon uptake.

## Introduction

Widescale greening/shrubification of the tundra is one of the most visible and ecologically important phenomena wrought by climate change in the Arctic^1–5^. Furthermore, there is evidence that tundra shrub abundance and cover have been increasing since at least the mid−20th century as a response to the warming climate^6,7^. Satellite data show positive trends in the normalized difference vegetation index (NDVI), a measure of greenness, across the arctic over the past few decades^8–11^. Strong positive correlations between NDVI, leaf area index (LAI)^12^ and biomass^13,14^ are well established for tundra ecosystems, such that this greening trend implies that vegetation cover and biomass have increased. In situ studies also support this conclusion^15–18^, and it has been estimated that above ground biomass in the circumpolar arctic tundra has increased by 19.8% from 1982 to 2010^19^. Similarly, atmospheric carbon dioxide (CO_2_) concentrations at high northern latitudes have shown enhanced seasonality since 1960^20,21^, thus showing a clear link between tundra greening and changes to tundra carbon cycling^22^. This enhanced seasonality is driven by increases in photosynthetic carbon uptake through changes in high latitude vegetation cover^21^. While greater leaf area in a canopy (LAI) intuitively should lead to greater photosynthesis and GPP, canopy architecture and self-shading complicate this^23,24^. In addition, there are many reasons to expect that increasing vegetation cover also affects ecosystem respiration, through both increased autotrophic respiration^25^ from increased foliar and root tissue^26^ and indirect effects on microbial respiration^27–29^. Thus, the impact of changing plant cover on the tundra carbon balance will depend on the balance between how much carbon is gained from increased photosynthesis and how much is lost from increased ecosystem respiration.

The impacts of increasing vegetation cover in the tundra occur concomitantly with rising temperatures. Thus, the distinct temperature responses of the physiological processes that drive the two components and competing fluxes of net ecosystem exchange (NEE), gross primary productivity (GPP) and ecosystem respiration (ER), must be considered. Photosynthesis in C_3_ plants has a broad temperature range optimum, implying that the effect of temperature fluctuations on photosynthetic rates of tundra species might be relatively small during typical peak growing season temperatures^30,31^. In contrast, rates of leaf respiration increase exponentially with temperature, peaking at a high critical temperature and then rapidly declining^32^. The respiratory peak occurs at a higher temperature than the photosynthetic optimum and falls outside the temperature ranges typically experienced in the tundra^32^. Similarly, rates of both root and microbial respiration also have an exponential response to temperature^25,33,34^. Because the optimum temperature for photosynthesis^35,36^ is lower than that for respiration^32^, at higher temperatures carbon uptake by plants decreases more rapidly than leaf respiration. While photosynthesis and respiration at the foliar level cannot be directly scaled up to GPP and ER, they can be indicative of how these ecosystem level carbon fluxes respond to temperature, suggesting that increases in ER will outpace GPP as ambient temperatures continue to rise and thereby decreasing the tundra’s overall carbon uptake. Two important questions follow: At what combination of light availability and temperature does this carbon sink-to-source threshold lie? And how is this threshold affected by increases in vegetation cover?

## Results and Discussion

To determine where this carbon sink-source threshold lies within current and near-future environmental conditions, we measured vegetation cover and carbon flux in two Alaskan tussock tundra ecosystems, which are generally not considered water limited^37^. One study region was located on the Seward Peninsula near Nome, and the other north of the Brooks Range, near Toolik Lake. The two sites differ in canopy cover, with the Toolik site having a greater average LAI (0.43 ± 0.02) than the Nome site (0.32 ± 0.01). Additionally, we set up fenced exclosures in Nome to exclude herbivore activity, thereby protecting the tundra from loss of vegetation cover and experimentally increasing LAI. All measurements were taken 1 year after fences were installed. At Nome, the exclosure treatment plots showed a moderately (16%) higher LAI compared to CT plots (${{{\rm{EX}}}}$ $\bar{x}=0.37\pm 0.01,{{{\rm{CT}}}}\bar{x}=0.32\pm 0.01$). Further, we found significant differences in the abundance of all growth forms, except forbs, between EX and CT (Supp. Figure 1). Despite these specific differences in growth form abundance, PERMANOVA analysis indicates that there was no significant difference in overall vegetation community composition between EX and CT. This finding enabled consideration of the effect of differences in LAI alone, without a confounding effect of differences in species composition, on the carbon sink-source threshold.

**Fig. 1:**
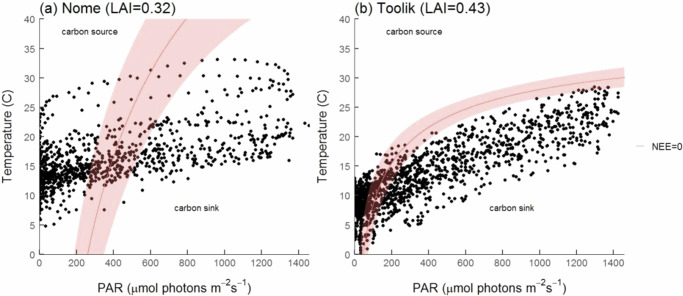
Temperature- and light-dependent carbon sink-source threshold for two tussock tundra sites. **a** Nome (LAI = 0.32) and (**b**) Toolik (LAI = 0.43). The solid line indicates the mean sink to source threshold (i.e. the combination of PAR and temperature at which NEE = 0) for all measured plots and the shaded area indicates the 95% confidence interval. The models are limited to temperatures above 0 °C. Symbols represent measured combinations of light and temperature at half hour intervals over the month of July 2019. Circles that fall above the threshold indicate the system is predicted to act as a carbon source, circles that fall below the threshold indicate a predicted carbon sink, and circles that fall within the shaded region of the threshold represent carbon neutral conditions.

At both Nome and Toolik, we made replicate measurements of NDVI and whole-ecosystem CO_2_ exchange (NEE) during the peak of the growing season (July). At the Nome sites, all measurements were made both within (EX) and outside of (CT) fenced exclosures (see methods). From these measurements, we derived the following additional variables: LAI, growth form abundance, ecosystem respiration (ER), gross primary productivity (GPP) and net ecosystem exchange (NEE). Measurements of CO_2_ exchange over a range of light levels were used to parameterize a model describing the response of NEE^38^ for each location. This was then used to calculate the temperature at which NEE = 0 *μ*mol carbon m^*−*2^ s^*−*1^ at a given light level. The resulting relationship represents a temperature- and light-dependent carbon sink-source threshold response (Fig. 1a). The curve can best be interpreted by considering the relative impacts of GPP and ER on NEE as light levels change. At low light levels NEE is dominated by ER as GPP is close to or below the light compensation point for photosynthesis. This means the ecosystem is above the sink-source threshold and is a source at almost all temperatures. As light level increases above the light compensation point for GPP, carbon uptake increases, and so the sink-source threshold occurs at progressively higher temperatures which are needed for ER to equal GPP. At high lights levels the GPP response begins to saturate, and so progressively smaller and smaller increases in temperature are required for ER to equal GPP. In order to demonstrate the ecological relevance of these sink-to-source thresholds, ambient light and air temperatures were recorded at half hour intervals during the peak of the growing season (July 2019) for the Nome and Toolik regions.

The Nome and Toolik tussock-tundra ecosystem plots differed in the shape of the PAR-temperature relationship describing the sink-to-source threshold (i.e. where NEE = 0; Fig. 1). During July at Nome (Fig. 1a), 40% of the half-hourly recorded measurements of light+temperature were above the sink-source threshold, and thus we predict the plots would act as a carbon source, and 26% were below the threshold (predicted carbon sink) (Table 1). All instances of light+temperature recordings crossing the sink-source threshold into the carbon source region occurred below ~500 μmol photons m^−2^ s^−1^. At Toolik, the sink-source threshold was within the range of currently experienced environmental conditions at lower light levels and temperatures than at Nome (Fig. 1b). During July, 25% of the recorded environmental conditions were above the sink-source threshold (predicted carbon source), and 53% were below the threshold (predicted carbon sink). All instances of light+temperature recordings exceeding the sink-source threshold occurred below 200 μmol photons m^−2^ s^−1^, which, while low, represent 47% of all recorded environmental data. Moreover, the mean light level for the month of July (peak growing season) was only 380.4 ± 10.2 μmol photons m^−2^ s^−1^, and the median light level was only 224.5 μmol photons m^−2^ s^−1^. Taken together, it is possible that moderate increases in temperature at relatively low light levels could result in conditions that either meet or surpass the carbon sink-source threshold for tussock tundra ecosystems, causing net source activity to be reached or surpassed more frequently than might be predicted under the less common local conditions of high temperatures and light.

**Table 1:** Comparison of leaf area index and the percentage of active season (July) that tussock tundra communities are predicted to be a sink, source or neither (within the 95% CI)

Community	Leaf area index (m^2^ m^−2^)	Sink (%)	Source (%)	Within 95% CI (%)
Nome (control)	0.32^a^ (±0.01)	26.1	40.4	33.5
Nome (exclosure)	0.37^b^ (±0.02)	24.2	19.5	56.3
Toolik	0.43^c^ (±0.02)	53.0	24.9	22.0

**Fig. 2:**
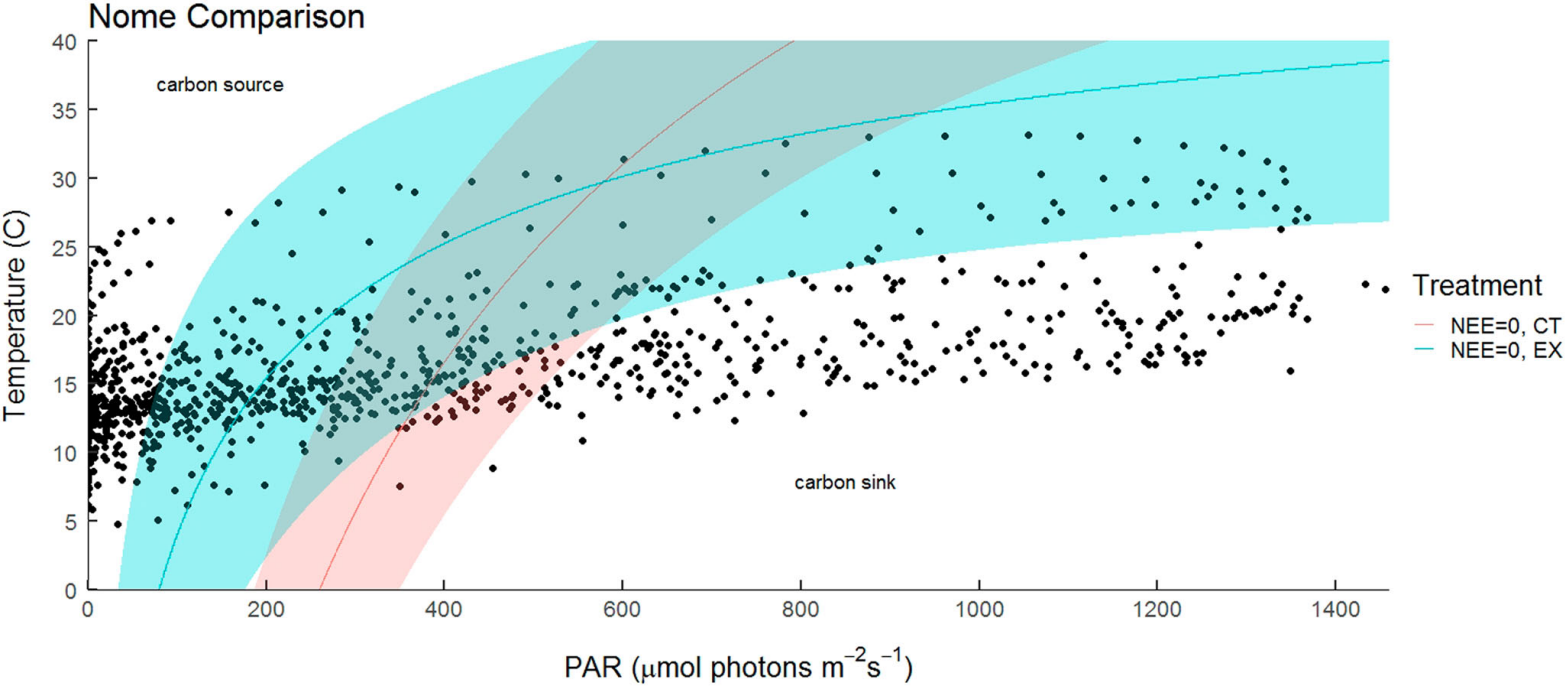
Temperature and light dependent carbon sink-source threshold for control and exclosure tussock tundra sites at Nome. The solid line indicates the mean sink-source threshold (i.e. the combination of PAR and temperature at which NEE = 0) for each treatment and the associated shaded area indicates the 95% confidence interval for each treatment. Red denotes control plots (LAI = 0.32) and green denotes exclosure plots (LAI = 0.37). The models used are limited to temperatures above 0 °C. Black symbols represent light and temperature conditions at half hour intervals over the period of 7/9-7/31 2019 for which data was available. Circles that fall above the threshold indicate the system is predicted to act as a carbon source, circles that fall below the threshold indicate a predicted carbon sink, and circles that fall within the shaded region of the threshold represent carbon neutral conditions. Regions where the two sink-source threshold responses differ significantly are indicated by the yellow shading.

Predictions based on the temperature- and light-dependent carbon sink-source thresholds for the various tussock tundra communities described in Figs. 1 and 2. For leaf area index values, means with different superscripts denote that they are significantly different at *p* < 0.05.

Another potential implication of the findings in Fig. 1 is the impact of vegetation cover (LAI) on the response. In comparison to Nome (0.32 ± 0.01), the higher LAI tussock-tundra at Toolik (0.43 ± 0.02) maintained net sink activity at significantly lower incident light levels. It also had a response curve with a relatively higher temperature sink-source threshold at low light, but a much lower temperature threshold at high light (the responses overlap and did not differ significantly over the PAR range ~300–650 μmol photons m^−2^ s^−1^). We also confirmed this LAI effect experimentally at Nome, where the exclosure treatment plots showed a 16% higher LAI compared to CT plots $\left({{{\rm{EX}}}}\bar{x}=0.37\pm 0.01,{{{\rm{CT}}}}\bar{x}=0.32\pm 0.01\right)$. Similar to the findings in Fig. 1, we found a difference in the shape of the sink-source threshold response between the higher (EX) and lower LAI (CT) plots, and once again the curves overlapped, with the difference in response significant only below ~370 μmol photons m^−2^ s^−1^ and above ~850 μmol photons m^−2^ s^−1^ (Fig. 2). Under low light conditions, higher temperatures and lower light levels were required for the sink-source threshold to be surpassed in the high compared with the low LAI plots. This response was reversed at high light levels beyond 895 μmol photons m^−2^ s^−1^, where the temperatures required to cross the sink-source threshold were lower in the high cover EX plots than the low cover CT plots. Accordingly, in the lower LAI CT plots, 40.4% of the half-hourly recorded measurements of light+temperature during the growing season were above the sink-source threshold (predicted source), compared with 19.5% for the higher LAI EX plots (Table 1). The mean light level at Nome during July was 355 ± 12 μmol photons m^−2^ s^−1^ while the median was 203 μmol photons m^−2^ s^−1^, again highlighting the importance of low-light carbon dynamics in tundra ecosystems. Under high-light conditions (above ~850 μmol photons m^−2^ s^−1^), all instances of light+temperature recordings fell below the sink-to-source threshold in the low LAI plots. In contrast, 23.6% of light+temperature recordings lay within the 95% CI in the higher cover EX plots, though none lay above the threshold.

Interpretation of changes in the shape of the source-to-sink threshold responses seen above is complicated as it requires consideration of the relative importance of GPP and ER to NEE at different light levels, temperatures and LAIs. In the initial part of the response, high LAI canopies have larger light use efficiencies than low LAI canopies, which allows GPP to compensate for increasing ER from increasing temperature. As light level increases, GPP begins to saturate and so temperature increases will lead to greater changes in ER relative to GPP. The reason that higher LAI communities cross the sink-to-source threshold at lower temperatures under high light than lower LAI communities is due to increases in self-shading from a denser canopy^12^. Under direct light, the proportion of canopy leaves in deep shade is nearly triple that under diffuse light conditions^23^. Thus, despite increases in vegetation cover leading to greater potential GPP through an increase in photosynthetic surface area^12^, increases in actual GPP will be less at progressively higher LAI than those at lower LAI. In addition, a high LAI canopy will likely have a more sensitive thermal response of ER (greater *Q*_10_) relative to a low LAI canopy. Canopies with higher LAI are also associated with indirect factors that increase respiration. For example, the fine root carbon pool has been shown to increase linearly up to an LAI of 1 m^2^/m^2^ (which both of our study regions fall well below)^26^ leading to an increase in the autotrophic respiration portion of ecosystem respiration. Because it has been estimated that autotrophic respiration can be responsible for between 35 and 90 percent of arctic tundra soil respiration^25,39^, even small increases in vegetation cover can have significant effects on ER as vegetation cover increases. In addition, organic carbon inputs to the soil (e.g. root exudates and litter deposition) increase the substrate pool that supports microbial activity, affecting both soil microbial metabolism^27^ and microbial diversity^28^. In this way, changes in vegetation cover can also exert influence on the heterotrophic portion of ER. The impacts of vegetation cover on ER would be most apparent under higher temperatures as the ER temperature response is exponential^38^.

Our findings have a number of potential implications:

1. Tundra ecosystems with different canopy cover (LAI) may have quite different sink-source threshold profiles. We found that under low light, low LAI tundra is more influenced by small increases in temperature relative to the response under moderate-high level light levels, but that this effect is partially offset by increases in vegetation cover in tundra with higher LAI. This result could be significant, given that the Arctic is expected to become cloudier during the summer^40^ and reinforces the importance of carbon flux dynamics under lower light. Importantly, the degree to which increasing cloudiness would push ecosystems to become carbon sources may depend on LAI.
2. Because of the shape of the temperature- and light-dependent carbon sink-source threshold response, warming may tend to drive greater source activity in those sites with higher canopy cover, which transition from a carbon sink to source under lower temperatures at high light levels than lower vegetation cover tundra ecosystems.
3. As tundra ecosystems ‘green’ in response to warming, their sink-source threshold profiles may change. As tundra ecosystems experience rising temperatures, our results suggest that the concomitant greening may elevate the temperatures at which tussock-tundra turns into a carbon source under commonly experienced moderate-low light conditions, increasing the potential to act as a carbon sink. However, while arctic greening might partially offset the impacts of warming at low light levels, increasing cover may also increase the vulnerability of tussock-tundra ecosystems crossing the sink-source threshold under increasingly frequent high light and high temperature conditions.
4. Linking 1, 2 and 3, the net impact of tundra greening will likely depend on the degree of warming and light level/cloud cover. Taken together, progressive warming and associated greening have the synergistic potential to push tundra ecosystems up to, and beyond, the threshold at which they switch from a carbon sink to a carbon source more often during the growing season. Climate change is also predicted to increase temperature variance^41,42^ which in turn contributes to higher frequencies of extreme climatic events including heat waves^43,44^. Our results from this small-scale plot-level study suggest that the negative effects of increasingly common high temperature events on overall carbon uptake during the growing season may also be exacerbated by increases in vegetation cover. Better elucidation of the complex impact of these drivers to short-term (environmental) and long-term (patterns of disturbance) carbon dynamics should be the focus of future study. This work should include a wider range of sites/years and capture community responses at greater scale (e.g. eddy covariance measurements to inform process-based understanding). This will not only provide improved estimates of net carbon uptake but will also allow for more nuanced predictions of how tundra NEE is affected by the direct and indirect consequences of Arctic warming.

Land-atmosphere carbon exchange is most active during the Arctic tundra’s growing season and carbon uptake by photosynthesis is limited to this period^45^. Thus, understanding which conditions enhance or reduce carbon uptake during arctic summers provides insight into the tundra’s overall carbon balance. It is common to report the effects of differing environmental and management treatments on net ecosystem exchange at 600 μmol photons m^−2^ s^−1^^12,23,46^, but our analysis indicates that this light level lies in the region that may not display any significant difference between treatments (especially in relation to impacts on LAI). This suggests that limiting reporting to this particularly light level may obscure treatment effects with real-world relevance. Previous studies have reported that, in response to warming, ecosystem carbon turnover increases because of increases in both gross primary productivity and ecosystem respiration, with little to no impact on overall carbon balance of the ecosystem^47,48^. Our findings provide important insight into why, and under what specific environmental conditions, this might change in a warming world, especially at sites with different underlying levels of canopy cover, and those experiencing progressive greening.

## Methods

### Study Site and Experiment Set Up

Three replicate sites were established in tussock tundra near Nome, AK (65.1°N, 164.7°W 6 m a.s.l.) and Toolik Lake, AK (68.6°N, 149.5°W, 760 m a.s.l.) for a total of 6 sites. At Nome, we set up an 8 m x 8 m fenced exclosure with a 0.635 cm square mesh, to prevent mammalian herbivore grazing and increase vegetation cover, and identified an 8 m x 8 m unfenced control plot marked at each corner with pin flags at each site. Within each treatment at each site, three 0.75 m circular subplots were randomly chosen for ecosystem flux measurements (for a total of 9 subplots at Toolik and 18 subplots at Nome). All subplots were located at least 0.5 m away from the borders of each plot to avoid edge effects due to differences in snow accumulation immediately next to the fences on our measurements and disturbance due to walking on the tundra. We measured each subplot for vegetation abundance, NDVI, and carbon flux. All data were collected during the month of July 2019.

### NDVI

The normalized difference vegetation index (NDVI) has been shown to correlate with leaf area index (LAI) in a variety of different ecosystems, including arctic tundra^12^. We measured NDVI at each subplot using a RapidSCAN CS-45 (Holland Scientific, Lincoln, NE). The RapidSCAN was held 1 m above the center point of each circular flux plot, and NDVI was logged 6-8 times as the RapidSCAN was rotated about the center point for the instrument’s 0.1 m by 0.8 rectangular footprint to cover the entire flux plot. As the previously published tundra NDVI-LAI relationship^12^ was developed with different instrumentation, we needed to calibrate our RapidSCAN data as the raw NDVI values recorded by RapidSCAN were slightly lower than NDVI values measured via the Unispec (PP Systems, Haverhill, MA) which was used by Street et al. ^12^.

We cross-calibrated the two instruments by taking measurements with both instruments along a nutrient gradient at an existing long-term experiment near Toolik Field station to provide an LAI gradient. NDVI was calculated from Unispec data using the following equation:

1 $${NDVI}=\frac{{R}_{{NIR}}-{R}_{{VIS}}}{{R}_{{VIS}}+{R}_{{VIS}}}$$

where R_NIR_ is the reflectance at 0.725−1.0 µm and R_VIS_ is the reflectance at 0.56–0.68 µm. The relationship between the two instruments was: NDVI Unispec = NDVI Rapidscan * 0.53 + 0.29, R^2^ = 0.66. The converted Rapidscan NDVI values were subsequently used to estimate LAI using the model described for tussock tundra^12^:

2 $${LAI}=0.0064{e}^{7.21* {NDVI}}$$

### Vegetation abundance

We measured vegetation abundance at each subplot using the point-frame method^49^. The circular pointframe was 80 cm in diameter and divided into a grid with points marked every 10 cm for a total of 62 points. We dropped a long pin at each point and recorded any vegetation by species name (or growth form) that touched the pin. Species were then categorized by growth forms: moss, dwarf deciduous shrubs, dwarf evergreen shrubs, forbs, graminoids and lichen.

### Ecosystem CO_2_ exchange measurements

To measure ecosystem CO_2_ exchange, we attached a custom cylindrical chamber^46^ made of clear polycarbonate with a clear lid (0.75 m in diameter and 0.31 m in height) to a Li-6400XT (IRGA, Li-Cor, Lincoln, NE) infrared gas analyzer. The system recorded CO_2_ and water vapor concentration changes in the chamber, and photosynthetically active radiation (PAR) and air temperature within the chamber over an interval of 40 s after stabilization of environmental conditions. Ambient light reaching the top of the canopy was measured as the photosynthetic photon flux density using a Li−190 quantum sensor (LiCor, Lincoln NE, USA) attached to the surface of the flux chamber and recorded by the gas-exchange system. Air temperature inside the chamber was also digitally recorded by the gas exchange system using a fine wire (30 gauge) type E thermocouple (5SRTC-KK-E-30-72, Omega Engineering Inc, Norwalk, CT, USA) placed within the upper canopy and out of direct sunlight. The air in the chamber was well mixed with two 16 cfm blowers attached to opposites sides of the chamber and directing the chamber air in a slight upward and center direction creating a circular airflow pattern (COM−11270, SparkFun electronics, Niwot Colorado, USA). The chamber was fitted with a thick plastic skirt attached at the bottom. We placed the chamber over each subplot using a heavy chain to weigh down the skirt and create a seal against the ground to minimize gas leaks. We measured gas flux for each subplot for at least 4 different light levels, ranging from full sun to complete darkness, with a minimum of three measurements per light level. Light levels were manipulated by covering the chamber with shade cloths of varying thicknesses, leaving the chamber exposed to full sun, or covering the chamber with a blackout cloth for dark measurements. Measurements with poor quality data such as unstable PAR or obvious leaks (e.g. negative NEE during dark measurements) were discarded. Each measurement was then used to calculate Net Ecosystem Exchange (NEE) (μmol m^−2^ s^−2^) under ambient PAR and temperatures for each subplot using Eq. 3

3 $${NEE}=\frac{\rho * V* \frac{{dC}}{{dt}}}{A}$$

The change in CO_2_ concentration, adjusted for dilution by water vapor, is dC/dt. Air density (ρ) is calculated from P/(RT) where P is pressure, R is the universal gas constant and T is temperature in K. V is the volume of the chamber (0.14 m^3^) and A is the surface area the chamber covers (0.435 m^2^). A total of 162, 209 and 159 NEE measurements were made at Toolik, Nome (CT) and Nome (EX), respectively, to parameterize the model as described below.

### Modeled NEE

We applied the PLIRTLE model, developed by Shaver et al. ^38^ with modifications detailed in Min et al. ^46^, to model CO_2_ fluxes in the arctic tundra, to our measured NEE data to analyze how the vegetation community, LAI and environmental conditions affect NEE in these two regions. NEE is defined as ecosystem respiration (ER) minus gross primary photosynthesis (GPP). We aggregated all NEE measurements per subplot within a treatment per region to parameterize the adapted version of the PLIRTLE model. ER measurements (CO_2_ flux measurements conducted in the dark) were aggregated across all subplots at Toolik and across all subplots within a treatment at Nome in order to cover a larger temperature range. We modeled ER first and then used the resulting parameter estimates combined with NEE measurements to model NEE for each subplot.

4 $${NEE}={ER}-\frac{{P}_{max \, L}* {LAI}* {E}_{0}* {PAR}}{{P}_{max \, L}+{E}_{0}* {PAR}}$$

The estimated parameters *P*_maxL_ (μmol CO_2_ m^−2^ leaf s^−1^), and E_0_ (μmol CO_2_ μmol^−1^ photons) refer to the theoretical light saturated photosynthesis rate and the light use efficiency. We used the dark gas flux measurements to parameterize the ER model described in equation 6 in Shaver et al. ^38^ (Eq. 5 below), where *R*_0_ (μmol m^−2^_leaf_ s^−1^), *R*_x_ (μmol m^−2^ ground s^−1^), and β (°C^−1^) are empirically derived parameters, and *T* is air temperature inside the chamber (°C). *R*_0_, *R*_x_ and β values were restricted to values ≥ 0.

5 $${ER}=\,({R}_{0}* {LAI}+{R}_{x}){e}^{\beta * T}$$

There were three subplots at Nome where the NEE model failed to converge, two control subplots and one exclosure subplot. In these cases, we substituted parameters derived from aggregating NEE measurements from the relevant treatment. From these subplot-specific NEE model parameters, we calculated the temperature at which NEE equaled zero per light level ranging from the lowest to the highest recorded PAR, at 0.5 μmol photons m^−2^ s^−1^ increments during the period of available environmental data.

### Environmental data

We obtained environmental data, specifically photosynthetic photon flux density (μmol photons m^−2^ s^−1^) and air temperature, from two eddy covariance towers near our sites (one per region). Data were available for Nome from 2 pm 7/9/2019 to 11:30 pm 7/31/2019 at half hour intervals (total of 1075 points). At Toolik, data were available the entire month of July 2019 at half hour intervals (total of 1488 points).

### Statistics

We checked for normality of residuals in datasets for which statistical analyses were undertaken. We compared LAI per treatment at Nome using linear mixed-effects models (LMM) with treatment as a fixed effect and site as a random effect. *P* values for treatment significance were obtained from maximum likelihood estimation. To test for differences in vegetation growth forms, we used generalized linear mixed models (GLMM) and used maximum likelihood estimation to obtain *p*-values. To analyze overall differences in vegetation community composition between treatments, we used permutational multivariate analysis of variance (PERMANOVA), blocked by site. Temperature thresholds for each treatment at Nome were compared for each light level using LMMs and as above, treatment was treated as a fixed effect and site as a random effect. *P*-values for treatment significance were again obtained from maximum likelihood estimation. Comparison of a portion of the lowest PAR levels (< 250 μmol photons m^−2^ s^−1^) resulted in a singular fit, however we retained this statistical model as it was suited to our experimental design and so that analyses were consistent for the entire PAR range. A *p*-value of < 0.05 was considered significant for all analyses. All analyses were completed in R v 3.5.1 (R Core Team, 2014) and using the following R packages: lme4^50^, lmertest^51^, lsmeans^52^, multcomp^53^ and vegan^54^. Figures were created using the ggplot2 package^55^.

### Reporting summary

Further information on research design is available in the Nature Portfolio Reporting Summary linked to this article.

### Supplementary information

Supplementary Information

Reporting Summary

## Data Availability

The data that support the findings in this study are available from the following sources. CO_2_ flux data, NDVI data and vegetation composition data is available through the Arctic Data Center (https://arcticdata.io/catalog; 10.18739/A2CF9J82N, 10.18739/A27S7HT97, 10.18739/A2N00ZV96).
