# Supplementary material for: The give and take of Arctic greening: differential responses of the carbon sink-to-source threshold to light and temperature in tussock tundra may be influenced by vegetation cover
Source: Commun Biol. 2024 Aug 6;7:950. doi: 10.1038/s42003-024-06600-z (PMC11303680; doi:10.1038/s42003-024-06600-z)
Supplement: Supplementary file 1 — Supplementary Information [file 42003_2024_6600_MOESM1_ESM.pdf]

Supplementary information

Mean LAI for the Toolik region was 0.43 (±0.02). Vegetation abundances were recorded as pin hits as detailed in the methods. Graminoids ($\bar{x}$ = 35.20 ± 1.98), dwarf deciduous shrubs ($\bar{x}$ = 13.90±1.24), dwarf evergreen shrubs ($\bar{x}$ = 24.90 ± 1.66) and moss ($\bar{x}$ = 40.00±2.11) accounted for the majority of the vegetation. Forbs ($\bar{x}$ = 4.44±0.70) and lichen ($\bar{x}$= 3.89± 0.66) were present, but much rarer.

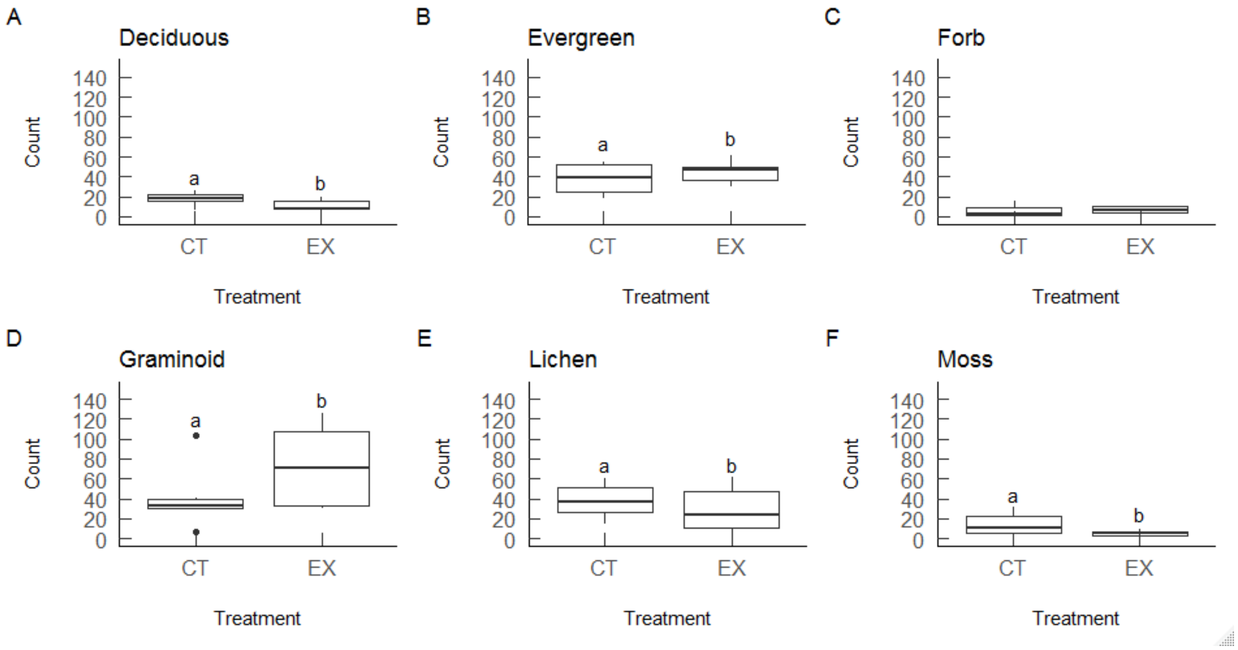

Supplementary Figure 1. Vegetation abundance in different growth forms (a-f) in tussock-tundra at Nome. Significant differences ($p<0.05$) between treatments are indicated by letters.

In the Nome region, mean LAI for control plots was 0.32 (±0.01) and for exclosure plots was 0.37 (±0.01). We observed a significantly higher abundance (as measured in pin hits) of dwarf evergreen shrubs (EX $\bar{x}$ = 46.10 ± 2.26, CT $\bar{x}$ =38.90 ± 2.08) and graminoids (EX $\bar{x}$= 75.80 ± 2.07, CT $\bar{x}$=38.70 ± 2.90) in EX compared to CT, while dwarf deciduous shrubs (EX $\bar{x}$ = 10.20 ± 1.07, CT $\bar{x}$=18.10 ± 1.42), lichens (EX $\bar{x}$=28.40 ± 1.78 CT $\bar{x}$=39.20 ± 2.09) and moss (EX $\bar{x}$ = 4.89 ± 0.74, CT $\bar{x}$ = 13.67 ± 1.23) were found to have a lower abundance in EX compared to CT. We did not find a significant difference in forb abundance between treatments.
