# Supplementary material for: The give and take of Arctic greening: differential responses of the carbon sink-to-source threshold to light and temperature in tussock tundra may be influenced by vegetation cover
Source: Commun Biol. 2024 Aug 6;7:950. doi: 10.1038/s42003-024-06600-z (PMC11303680; doi:10.1038/s42003-024-06600-z)
Supplement: Supplementary file 2 — Reporting Summary [file 42003_2024_6600_MOESM2_ESM.pdf]

nature portfolio

Corresponding author(s): Kevin L. Griffin
Last updated by author(s): Jun 6, 2024

Reporting Summary

Nature Portfolio wishes to improve the reproducibility of the work that we publish. This form provides structure for consistency and transparency in reporting. For further information on Nature Portfolio policies, see our Editorial Policies and the Editorial Policy Checklist.

Please do not complete any field with "not applicable" or n/a. Refer to the help text for what text to use if an item is not relevant to your study.
For final submission: please carefully check your responses for accuracy; you will not be able to make changes later.

Statistics

For all statistical analyses, confirm that the following items are present in the figure legend, table legend, main text, or Methods section.

n/a	Confirmed	
☐	☒	The exact sample size (*n*) for each experimental group/condition, given as a discrete number and unit of measurement
☐	☒	A statement on whether measurements were taken from distinct samples or whether the same sample was measured repeatedly
☐	☒	The statistical test(s) used AND whether they are one- or two-sided<br>***Only common tests should be described solely by name; describe more complex techniques in the Methods section.***
☐	☒	A description of all covariates tested
☐	☒	A description of any assumptions or corrections, such as tests of normality and adjustment for multiple comparisons
☐	☒	A full description of the statistical parameters including central tendency (e.g. means) or other basic estimates (e.g. regression coefficient) AND variation (e.g. standard deviation) or associated estimates of uncertainty (e.g. confidence intervals)
☐	☒	For null hypothesis testing, the test statistic (e.g. *F*, *t*, *r*) with confidence intervals, effect sizes, degrees of freedom and *P* value noted<br>***Give P values as exact values whenever suitable.***
☒	☐	For Bayesian analysis, information on the choice of priors and Markov chain Monte Carlo settings
☒	☐	For hierarchical and complex designs, identification of the appropriate level for tests and full reporting of outcomes
☐	☒	Estimates of effect sizes (e.g. Cohen's *d*, Pearson's *r*), indicating how they were calculated

Our web collection on statistics for biologists contains articles on many of the points above.

Software and code

Policy information about availability of computer code

Data collection	LI-6400XT instrument (embedded) software v5.3.2
Data analysis	All analyses were completed in R v 3.5.1 (R Core Team, 2014) and using the following R packages: lme4 (Bates et al. 2015), lmertest (Kuznetsova et al. 2017), lsmeans (Lenth 2016), multcomp (Hothorn et al. 2008) and vegan (Oksanen et al. 2008). Figures were created using the ggplot2 package (Wickham 2016).

For manuscripts utilizing custom algorithms or software that are central to the research but not yet described in published literature, software must be made available to editors and reviewers. We strongly encourage code deposition in a community repository (e.g. GitHub). See the Nature Portfolio guidelines for submitting code & software for further information.

Data

Policy information about availability of data

All manuscripts must include a data availability statement. This statement should provide the following information, where applicable:

- Accession codes, unique identifiers, or web links for publicly available datasets
- A description of any restrictions on data availability
- For clinical datasets or third party data, please ensure that the statement adheres to our policy

The data that support the findings in this study are available from the following sources. CO2 flux data, NDVI data and vegetation composition data will be available through the Arctic Data Center (https://arcticdata.io/catalog; DOI pending).

Research involving human participants, their data, or biological material

Policy information about studies with human participants or human data. See also policy information about sex, gender (identity/presentation), and sexual orientation and race, ethnicity and racism.

Reporting on sex and gender	*Use the terms sex (biological attribute) and gender (shaped by social and cultural circumstances) carefully in order to avoid confusing both terms. Indicate if findings apply to only one sex or gender; describe whether sex and gender were considered in study design; whether sex and/or gender was determined based on self-reporting or assigned and methods used. Provide in the source data disaggregated sex and gender data, where this information has been collected, and if consent has been obtained for sharing of individual-level data; provide overall numbers in this Reporting Summary. Please state if this information has not been collected. Report sex- and gender-based analyses where performed, justify reasons for lack of sex- and gender-based analysis.*
Reporting on race, ethnicity, or other socially relevant groupings	*Please specify the socially constructed or socially relevant categorization variable(s) used in your manuscript and explain why they were used. Please note that such variables should not be used as proxies for other socially constructed/relevant variables (for example, race or ethnicity should not be used as a proxy for socioeconomic status). Provide clear definitions of the relevant terms used, how they were provided (by the participants/respondents, the researchers, or third parties), and the method(s) used to classify people into the different categories (e.g. self-report, census or administrative data, social media data, etc.) Please provide details about how you controlled for confounding variables in your analyses.*
Population characteristics	*Describe the covariate-relevant population characteristics of the human research participants (e.g. age, genotypic information, past and current diagnosis and treatment categories). If you filled out the behavioural & social sciences study design questions and have nothing to add here, write "See above."*
Recruitment	*Describe how participants were recruited. Outline any potential self-selection bias or other biases that may be present and how these are likely to impact results.*
Ethics oversight	*Identify the organization(s) that approved the study protocol.*

Note that full information on the approval of the study protocol must also be provided in the manuscript.

Field-specific reporting

Please select the one below that is the best fit for your research. If you are not sure, read the appropriate sections before making your selection.

☐ Life sciences ☐ Behavioural & social sciences ☒ Ecological, evolutionary & environmental sciences

For a reference copy of the document with all sections, see nature.com/documents/nr-reporting-summary-flat.pdf

Ecological, evolutionary & environmental sciences study design

All studies must disclose on these points even when the disclosure is negative.

Study description	Tussock tundra carbon flux measurements were made over a range of ambient temperature and light conditions to assess ecosystem carbon balance and source sink transitions
Research sample	Replicate plot level carbon flux measurements from triplicate control and enclosure plots that varied in leaf area index at two tundra locations (near Toolik Lake Ak and near Nome AK)
Sampling strategy	sample size maximized spatial sampling and temporal proximity to create robust estimates of carbon flux across a range of temperature and light conditions.
Data collection	Data were automatically recorded by the instruments used as programmed by the authors.
Timing and spatial scale	Each flux measurement was begun immediately once statistical stability in carbon and water flux along with ambient light conditoins was achieved (as assessed automatically by the instruments used). Each flux measurement ended approximately 90 seconds later to avoid overheating of the chamber. The chamber was completely removed between measurements to allow for temperature equilibration with the ambient environment. All measurements were made over a two week window to avoid significant changes in the vegetation.
Data exclusions	Data were excluded due to a lack of light stabilit, the presence of obvious leaks (which can be detected by a change in pressure), or due to overheating (a change of more than 2.5 C during the 90 min measurement).
Reproducibility	The experiment was piloted the year before it was completed and the preliminary measurements were compared to the full experiment. Many replicate measurements were made in each plot over the course of two weeks.
Randomization	All measurements were made by the same group of authors (primarily Min and Griffin) with assistance from others.

Blinding	Field day represent raw instrument values and not processed fluxes and thus the researchers were blind to the final outcome

Did the study involve field work? ☒ Yes ☐ No

Field work, collection and transport

Field conditions	The experimental design was meant to capture the full range of ambient environmental conditions
Location	Three replicate sites were established in tussock tundra near Nome, AK (65.1^{o}N, 164.7^{o}W 6 m a.s.l.) and Toolik Lake, AK (68.6^{o}N, 149.5^{o}W, 760 m a.s.l.) for a total of 6 sites.
Access & import/export	Permits for this research were obtain either from the BLM or the land owners
Disturbance	The experiment was not destructive. The only damage was from walking on the tundra to access the plots. All measrments were made in a confined area set aside for gas exchange measurements to minimize any disturbance to other parts of the plots used for other project measurements.

Reporting for specific materials, systems and methods

We require information from authors about some types of materials, experimental systems and methods used in many studies. Here, indicate whether each material, system or method listed is relevant to your study. If you are not sure if a list item applies to your research, read the appropriate section before selecting a response.

Materials & experimental systems

n/a	Involved in the study	
☒	☐	Antibodies
☒	☐	Eukaryotic cell lines
☒	☐	Palaeontology and archaeology
☒	☐	Animals and other organisms
☒	☐	Clinical data
☒	☐	Dual use research of concern
☐	☒	Plants

Methods

n/a	Involved in the study	
☒	☐	ChIP-seq
☒	☐	Flow cytometry
☒	☐	MRI-based neuroimaging

Dual use research of concern

Policy information about dual use research of concern

Hazards

Could the accidental, deliberate or reckless misuse of agents or technologies generated in the work, or the application of information presented in the manuscript, pose a threat to:

No	Yes	
☒	☐	Public health
☒	☐	National security
☒	☐	Crops and/or livestock
☒	☐	Ecosystems
☒	☐	Any other significant area

Experiments of concern

Does the work involve any of these experiments of concern:

No	Yes	
☒	☐	Demonstrate how to render a vaccine ineffective
☒	☐	Confer resistance to therapeutically useful antibiotics or antiviral agents
☒	☐	Enhance the virulence of a pathogen or render a nonpathogen virulent
☒	☐	Increase transmissibility of a pathogen
☒	☐	Alter the host range of a pathogen
☒	☐	Enable evasion of diagnostic/detection modalities
☒	☐	Enable the weaponization of a biological agent or toxin
☒	☐	Any other potentially harmful combination of experiments and agents

Plants

Seed stocks	no plant materials were collected. Plant properties were measured in the field. These were all native plants growing naturally at the field sites.
Novel plant genotypes	none
Authentication	none

This checklist template is licensed under a Creative Commons Attribution 4.0 International License, which permits use, sharing, adaptation, distribution and reproduction in any medium or format, as long as you give appropriate credit to the original author(s) and the source, provide a link to the Creative Commons license, and indicate if changes were made. The images or other third party material in this article are included in the article's Creative Commons license, unless indicated otherwise in a credit line to the material. If material is not included in the article's Creative Commons license and your intended use is not permitted by statutory regulation or exceeds the permitted use, you will need to obtain permission directly from the copyright holder. To view a copy of this license, visit http://creativecommons.org/licenses/by/4.0/
